# Complement Component C3 and Butyrylcholinesterase Activity Are Associated with Neurodegeneration and Clinical Disability in Multiple Sclerosis

**DOI:** 10.1371/journal.pone.0122048

**Published:** 2015-04-02

**Authors:** Shahin Aeinehband, Rickard P. F. Lindblom, Faiez Al Nimer, Swetha Vijayaraghavan, Kerstin Sandholm, Mohsen Khademi, Tomas Olsson, Bo Nilsson, Kristina Nilsson Ekdahl, Taher Darreh-Shori, Fredrik Piehl

**Affiliations:** 1 Department of Clinical Neuroscience, Neuroimmunology Unit, Karolinska Institutet, Stockholm, Sweden; 2 Division of Alzheimer Neurobiology Center, Department of Neurobiology, Care Sciences and Society, Karolinska Institutet, Stockholm, Sweden; 3 Division of Clinical Immunology, Department of Immunology, Genetics and Pathology, Uppsala University, Uppsala, Sweden; 4 School of Natural Sciences, Linnæus University, Kalmar, Sweden; Weizmann Institute of Science, ISRAEL

## Abstract

Dysregulation of the complement system is evident in many CNS diseases but mechanisms regulating complement activation in the CNS remain unclear. In a recent large rat genome-wide expression profiling and linkage analysis we found co-regulation of complement C3 immediately downstream of butyrylcholinesterase (BuChE), an enzyme hydrolyzing acetylcholine (ACh), a classical neurotransmitter with immunoregulatory effects. We here determined levels of neurofilament-light (NFL), a marker for ongoing nerve injury, C3 and activity of the two main ACh hydrolyzing enzymes, acetylcholinesterase (AChE) and BuChE, in cerebrospinal fluid (CSF) from patients with MS (n = 48) and non-inflammatory controls (n = 18). C3 levels were elevated in MS patients compared to controls and correlated both to disability and NFL. C3 levels were not induced by relapses, but were increased in patients with ≥9 cerebral lesions on magnetic resonance imaging and in patients with progressive disease. BuChE activity did not differ at the group level, but was correlated to both C3 and NFL levels in individual samples. In conclusion, we show that CSF C3 correlates both to a marker for ongoing nerve injury and degree of disease disability. Moreover, our results also suggest a potential link between intrathecal cholinergic activity and complement activation. These results motivate further efforts directed at elucidating the regulation and effector functions of the complement system in MS, and its relation to cholinergic tone.

## Introduction

Multiple Sclerosis (MS) is primarily an autoimmune disease of the central nervous system (CNS) characterized by chronic inflammation leading to demyelination, axonal damage and subsequent neurological deficits [1]. With time a majority of patients with relapsing remitting MS (RRMS) will convert to a secondary progressive (SPMS) disease course [1]. A minority of patients displays a progressive course already from disease onset; primary progressive MS (PPMS), which has been suggested to represent RRMS “amputated” from its relapsing-remitting phase [1, 2].

Arguably, absence of remission in late stage RRMS reflects failed recovery processes due to repeated attacks of inflammation leading to irreversible axonal damage, the main determinant of irreversible disability in MS patients [3]. Thus, the transition to SPMS occurs when the CNS can no longer compensate for the neuronal loss [4]. However, it may also be that worsening in relative absence of relapses and radiological signs of inflammation in progressive MS could involve other disease mechanisms than in the relapsing-remitting phase, which is characterized by a T cell dependent immune response [5–7]. The complement system, an important part of the innate immune system with a wide range of effects in multiple disease states in the CNS could play such a contributory role [8]. In fact, several recent studies suggest that certain complement proteins are dysregulated in MS. For instance, certain types of MS lesions displays prominent complement activation [9]. Moreover, elevated levels of plasma C3, C4, C4a, C1 inhibitor, factor H, and reduced levels of C9 have been found in MS patients [10–13], as well as increased cerebrospinal fluid (CSF) C4b and alterations in C3 [14–16].

CSF levels of the central complement component C3 have been shown to correlate with degree of neurological impairment in several neurodegenerative diseases [17], but any relation to disease severity in MS is unknown.

We have previously found that genetically determined susceptibility to neurodegeneration after a standardized nerve injury among inbred rat strains is associated with a more prominent local inflammatory response with elevated expression of the upstream complement proteins C1q and C3 [18]. By performing genome-wide expression profiling and linkage analysis in a large inbred rat strain intercross we found co-regulation of complement C3 immediately downstream of butyrylcholinesterase (BuChE), an enzyme that hydrolyses acetylcholine (ACh) [19]. BuChE has been proposed as a risk gene for sporadic Alzheimer´s disease (AD) [20] and we recently showed that such patients carrying a BuChE allele coding for a protein with reduced activity displays lower levels of glial activation markers [21]. In an early study, the activity of the two main ACh hydrolyzing enzymes, acetylcholinesterase (AChE) and BuChE, were determined in CSF and brain tissue in a small group of patients, including one MS patient that displayed reduced AChE, but not BuChE activity [22]. Neuropathological studies on MS brain tissue have found increased BuChE activity associated with microglia within MS lesions, which could contribute to the pro-inflammatory milieu [23], as well as an altered balance of choline acetyltransferase (ChAT), the ACh synthesizing enzyme, and AChE in the hippocampus [24]. However, in a recent study the activity of AChE in CSF was found not to differ between MS patients and controls [25].

The aim of this study was to determine and correlate CSF levels of C3 in MS patients and controls, to different disease measures, including a biomarker for nerve injury, as well as to activity of the two main ACh hydrolyzing enzymes.

## Material and Methods

### Clinical material

CSF samples were obtained during diagnostic workup or for diagnostic or other clinical purposes from patients attending the Neurology Clinic, Karolinska University Hospital, Solna. Written informed consent was obtained from all patients and the study was approved by the Regional Ethical Vetting Board of Stockholm (Diary Number: 2009/2107-31-2). Clinical examinations were performed by specialists in neurology, and all patients diagnosed with MS fulfilled the McDonald criteria [26]. All MS patients were evaluated clinically at time of sampling with the Expanded Disability Status Scale (EDSS) and the subsequent calculation of Multiple Sclerosis Severity Scale (MSSS). Two patients in the RRMS relapse group received disease modifying treatments at time of sampling, one interferon-β1a and one natalizumab (with 8 weeks wash out). Sixteen RRMS patients were in relapse defined as an increase of ≥1 EDSS point with duration of at least one week, not longer than 3 months before sampling, where systemic infection had been ruled out. The remaining RRMS patients were in remission, defined as a stable clinical and neuroradiological status ≥3 months prior to sampling. SPMS was defined as an initial relapsing-remitting disease course followed by >12 months of continuous worsening of neurological function (≥0.5 EDSS point) not explained by relapses. The control group consisted of patients with non-inflammatory neurological/psychiatric conditions (OND) with normal magnetic resonance imaging (MRI) scans and lacking signs of inflammatory activity in CSF in terms of pleocytosis or intrathecal IgG production; psychosis n = 6; functional paresthesia n = 4; peripheral mononeuropathy n = 3; lumbago = 1; epilepsy n = 1; vertigo n = 1; fatigue n = 1; syringomyelia n = 1). Two patients had received corticosteroids for their MS relapse; two months before and one week before sampling, respectively. The exclusion of the treated individuals did not significantly alter the results. For patient characteristics see Tables 1 and 2. CSF cell counts, immunoglobulin content and electrophoresis were performed at the time of sampling at the Department of Clinical Chemistry, Karolinska University Hospital according to clinical routines.

**Table 1:** Demographics of MS patients and controls.

**Groups**	***n***	**Mean age (SD)**	**Females (%)**	**Mean EDSS (range)**	**Mean disease duration at sampling (range)**
**OND**	18	30.4 (7.9)	55.6	N/A	N/A
**RRMS**	33	36.6 (9.6)	70.6	2.0 (0–4.5)	5.4 (0–19)
**-Relapse**	16	34.1 (8.5)	81	2.0 (0–4.5)	4.8 (0–16)
**-Remission**	17	39.1 (10.2)	65	1.9 (0–4)	6.0 (0.5–19)
**SPMS**	9	52.0 (10.1)	55.6	5.2 (4–6.5)	17 (4–25)
**PPMS**	6	50.3 (8.5)	33.3	4.2 (3.5–5.5)	3.8 (2–10)
**All MS**	48	41.2 (11.6)	64.6	3.0 (0–6.5)	7.4 (0–25)

OND = Other Neurological/psychiatric Diseases, RRMS = Relapsing-Remitting Multiple Sclerosis, SPMS = Secondary Progressive MS, PPMS = Primary Progressive MS, EDSS = Expanded Disability Status Scale.

**Table 2:** Characteristics of MS patients and controls.

**Diagnosis**	***n***	**C3 median (range) μg/L**	**NFL median (range) ng/L**	**BuChE activity median (range) nmol/min/ml**	**AChE activity median (range) nmol/min/ml**	**CSF Cells median (range) x10** ^6^ **/L**	
**OND**	18	1818.1 (826.9–3354)	212 (77–472.8)	6.0 (2.7–9.9)	9.8 (6.9–17.5)		N/A
**RRMS**	33	2227.9 (856.6–4249.8)	1297.2 (293.7–9162.1)	5.7 (3.4–9.3)	8.0 (1.8–17.5)		4 (0–21)
**-Relapse**	16	2273.8 (1245.7–3986.6)	2237.5 (722.7–9162.1)	6.1 (4.4–9.3)	8.3 (1.8–17.5)		6 (0–21)
**-Remission**	17	1964.4 (856.6–4249.8)	738 (293.7–2756.1)	5.4 (3.4–8.4)	7.7 (2.8–12.1)		4 (0–21)
**SPMS**	9	2623.3 (1983.3–4723.3)	909.9 (280.4–4049.5)	7.0 (4.7–9.4)	5.6 (3.8–8.0)		3 (0–7)
**PPMS**	6	4180.8 (2279.1–5030.3)	874.5 (644.3–2940.7)	5.6 (3.7–8.4)	8.2 (3.9–12.1)		7 (0–12)
**All MS**	48	2400.3 (856.6–5030.3)	1028.5 (280.4–9162.1)	6.0 (3.4–9.4)	7.3 (1.8–17.5)		4 (0–21)

### Quantification of protein and cholinesterase enzymatic activity

Complement protein C3 levels in 1:100 diluted CSF were analysed with a sandwich ELISA as previously described [27]. In brief, a C3 assay plate was coated with rabbit anti-C3c (A0062, Dako) diluted 1:3000. C3c recognizes C3, C3b, iC3b, and C3c. Detection of the bound C3/C3-fragments was carried out with biotinylated anti-C3c diluted 1:3200, followed by streptavidin-HRP diluted 1:500 (Amersham). The concentration of C3, in each sample was determined using DeltaSoft (BioMetallics Inc, Princeton NJ, USA) software. A positive control of pooled plasma from five blood donors was included, and a sample with known concentrations of C3 was used as a standard.

Protein levels of BuChE and AChE were quantified in CSF diluted 1:4 by using a custom made ELISA, as previously described [28].

The activities of BuChE and AChE were measured using a modified version of Ellman's colorimetric assay, using 5.0 mM butyrylthiocholine iodide (Sigma) or 0.5 mM acetylthiocholine iodide (Sigma) as substrate in the presence of 1.0 μM of the selective AChE inhibitor BW280C51 (Sigma) or 0.1 mM of the selective BuChE inhibitor ethopropazine (Sigma) [29]. Using these selective AChE and BuChE inhibitors at the specified final concentrations, a possible cross contamination of the AChE and BuChE activities is expected to be less than 5%. To effectively minimize any intra-assay variation due to storage and sample handling, all CSF samples were kept at -80C until the assay and all samples were simultaneously thawed in an ice-water bath. Samples were added in triplicates and run together on one 384-wells plate.

The MS patients included here had been genotyped with Immunochip [30], and allelic differences in the BCHE gene were determined based on the rs1803274 polymorphism.

NFL was determined with a commercial ELISA kit (UMAN Diagnostics AB) in undiluted CSF according to the manufacturer´s instructions.

All quantifications were performed blinded and patient allocation to the different groups was performed before analyses by a single rater based on reviews of clinical records.

### Statistical analysis

Significance levels were calculated using one-way ANOVA with Tukey's post-hoc tests in GraphPad Prism 5.0 (San Diego, CA). Correlations between the different protein levels was calculated using Pearson’s correlations assuming equal distribution depicted with simple regression plots. P < 0.05 was considered statistically significant. * = p < 0.05; ** = p < 0.01; *** = p < 0.001.

## Results

### Elevated CSF C3 levels in MS correlate with disease course, MRI lesions, disease severity and a biomarker of nerve injury

**Fig 1:**
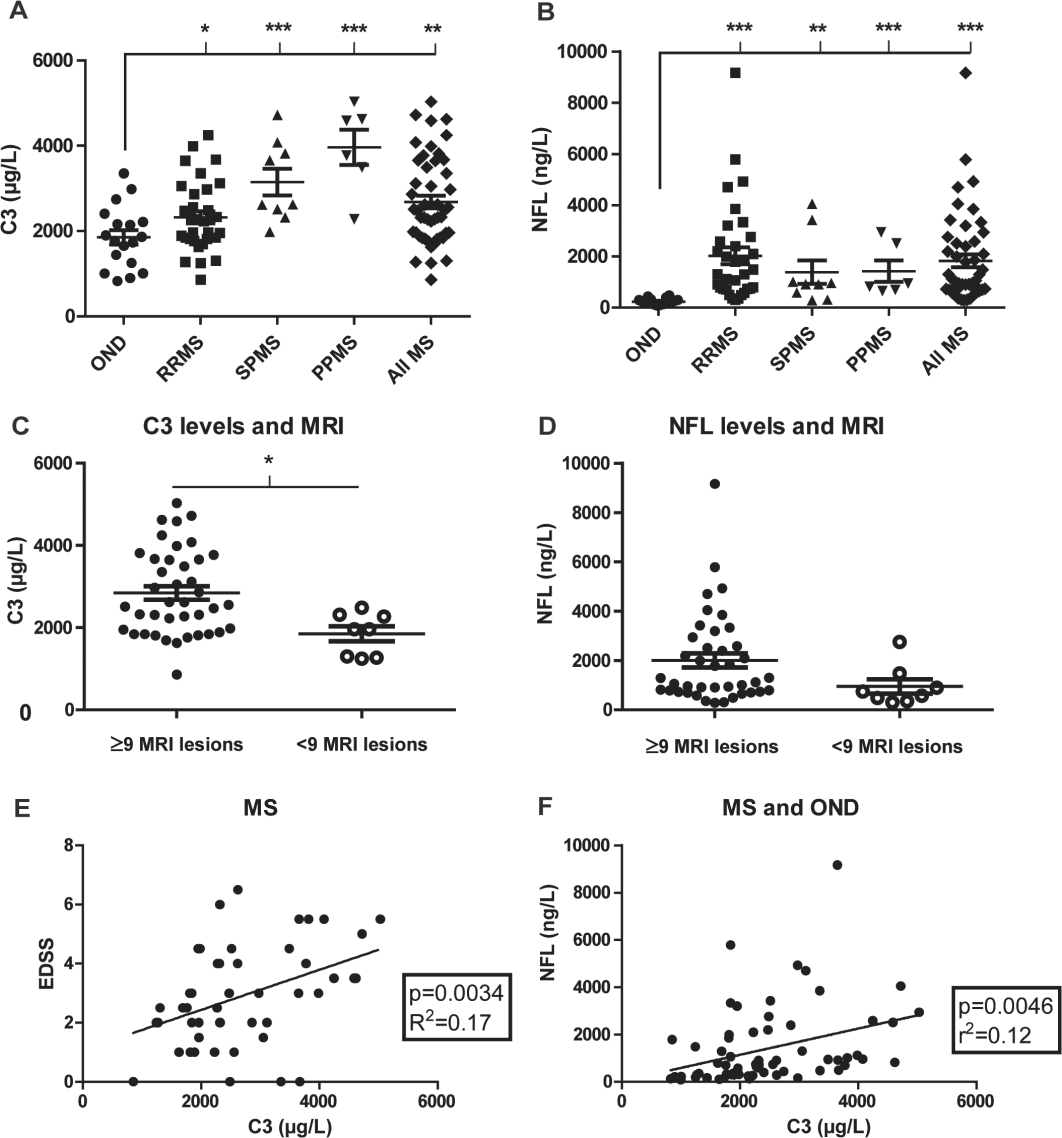
Levels of C3 in cerebrospinal fluid are increased in patients with MS and correlate with MRI lesions, clinical disability and levels of NFL. CSF was obtained from 48 patients diagnosed with RRMS, SPMS or PPMS and 18 controls with other neurological/psychiatric diseases (OND). Protein quantification demonstrates increased levels of C3 in the CSF of MS patients compared to controls, with higher levels in patients with progressive disease, especially PPMS (A). Levels of NFL, a marker of neurodegeneration, are also increased in MS patients compared to controls (B). Patients with 9 or more MRI lesions at the time of sampling have significantly higher C3 levels (C) and a non-significant increase in NFL levels (D). C3 levels correlate with degree of clinical disability as assessed with EDSS (E), and NFL (F). * = p < 0.05; ** = p < 0.01; *** = p < 0.001.

Levels of C3 and NFL, an established marker of neurodegeneration in MS [31–33], were analyzed in CSF obtained from a cohort of patients (n = 48) with relapsing-remitting (RRMS) or progressive (SPMS/PPMS) disease, as well as in controls with other non-inflammatory neurological/psychiatric diseases (OND; n = 18) (Fig 1A and 1B). For patient details see Tables 1 and 2. CSF NFL levels were significantly increased in all subgroups of MS compared to OND controls, with RRMS relapse > RRMS remission > SPMS = PPMS > OND (Table 2). Also the levels of C3 in CSF were significantly increased in MS compared to OND controls, but in a different rank order, with PPMS > SPMS > RRMS > OND (Table 2 and Fig 1A). Separate analysis of C3 and NFL levels were done on MS patients divided after ≥9 (n = 40) or <9 (n = 8) cerebral MRI lesions. MS patients that had ≥9 cerebral MRI lesions at the time of sampling also had significantly increased C3 levels compared to those with few MRI lesions (P = 0.011; Fig 1C). The same trend was seen with NFL, however not significant (P = 0.118; Fig 1D). In the MS cohort C3 levels correlated with disease severity as assessed by EDSS (Fig 1E) and to a lesser degree to MSSS (p = 0.023, R^2^ = 0.13). The correlation between C3 and NFL in the MS cohort was not significant (p = 0.11, R^2^ = 0.053), but became significant when the OND group was included in the analysis (Fig 1F). In contrast, there was no correlation between C3 and disease duration (p = 0.71, R^2^ = 0.0029), intrathecal antibody production (IgG index) (p = 0.35, R^2^ = 0.019) or CSF cell numbers (p = 0.81, R^2^ = 0.0012). There was a trend for a correlation between C3 and age in RRMS (p = 0.069, R^2^ = 0.1025), but not in OND (p = 0.77, R^2^ = 0.005).

### Levels of BuChE, but not AChE correlate with CSF C3 and NFL levels in MS patients

**Fig 2:**
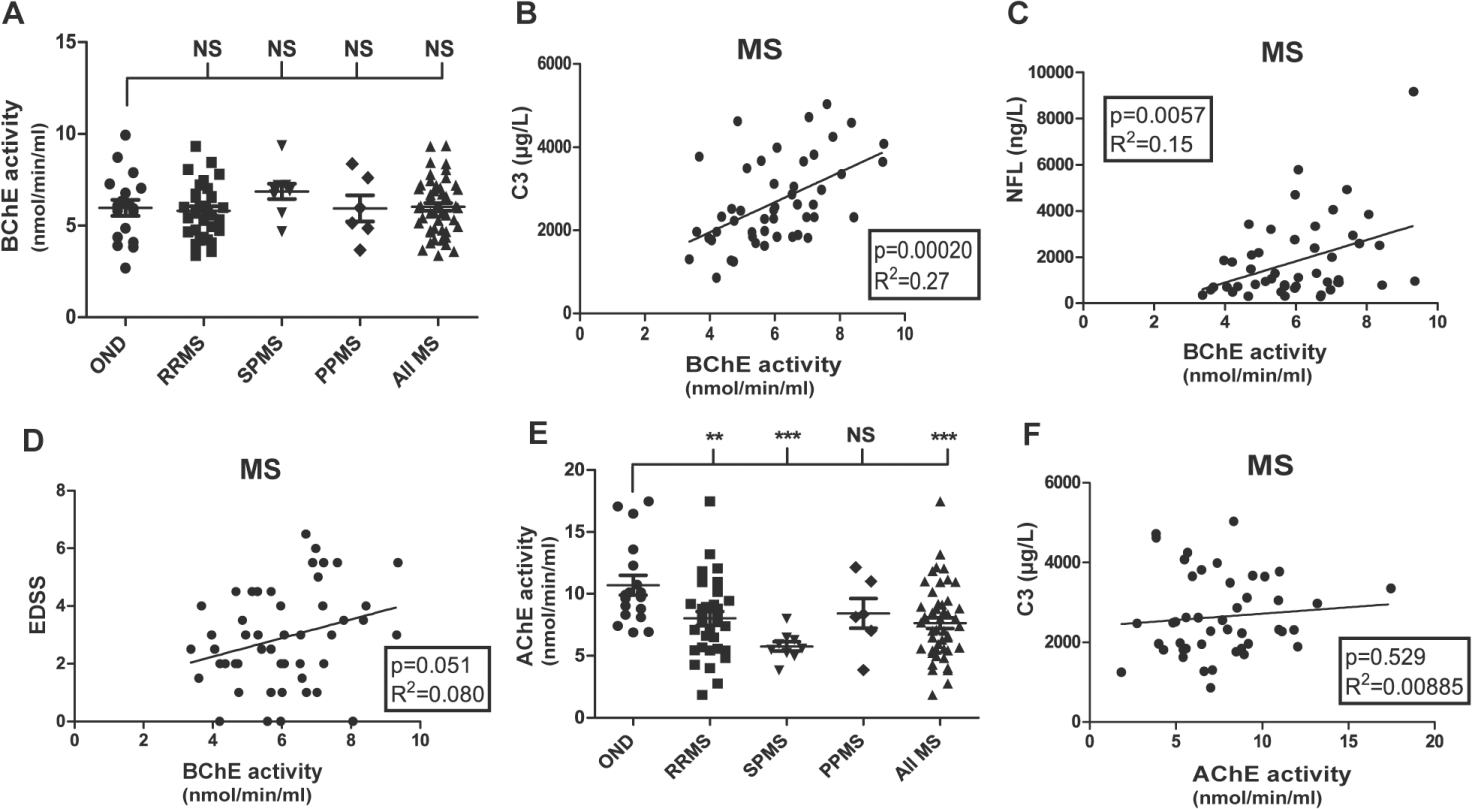
BuChE activity, but not AChE activity, correlates with levels of C3 and NFL. BuChE activity was not different between MS patients and controls at the group level, however, with a large spread within groups (A). A highly significant correlation between BuChE activity and C3 levels is evident (B). Also, BuChE activity correlated with levels of NFL (C) and demonstrates a close to significant trend for correlation to EDSS (D). Interestingly, AChE activity differs between MS patients and controls (E), but lacks correlation to C3 at the individual level (F). *p = < 0.05; ** = p < 0.01; *** = p < 0.001.

The genetic link between local expression of BuChE and C3 after experimental nerve injury provided a rationale to determine BuChE activity in the clinical samples [19,34]. In addition, activity of AChE, the other main ACh hydrolyzing enzyme [35], was determined. Enzymatic activity and protein levels displayed a high degree of correlation for BuChE (p<0.0001, R2 = 0.887). At the group level, BuChE activity in MS patients did not differ from controls (Fig 2A). However, in individual samples BuChE activity significantly correlated to both C3 and NFL in the MS group (Fig 2B and 2C), and displayed a trend for correlation to EDSS (Fig 2C and 2D). In contrast, AChE enzymatic activity at the group level was significantly decreased in MS patients as compared to OND (Fig 2E). On an individual level AChE activity did not correlate neither to C3 nor NFL (Fig 2F). On the contrary, there was a trend for a negative correlation between AChE activity and EDSS (p = 0.086, R2 = 0.064), thus, opposite to the pattern of BuChE. MS patients carrying the BuChE-K allele displayed an 8% lower BuChE activity than non-carriers, however, the difference was not statistically different.

### RRMS clinical relapse activity increase CSF NFL levels, but not BuChE activity or C3

**Fig 3:**
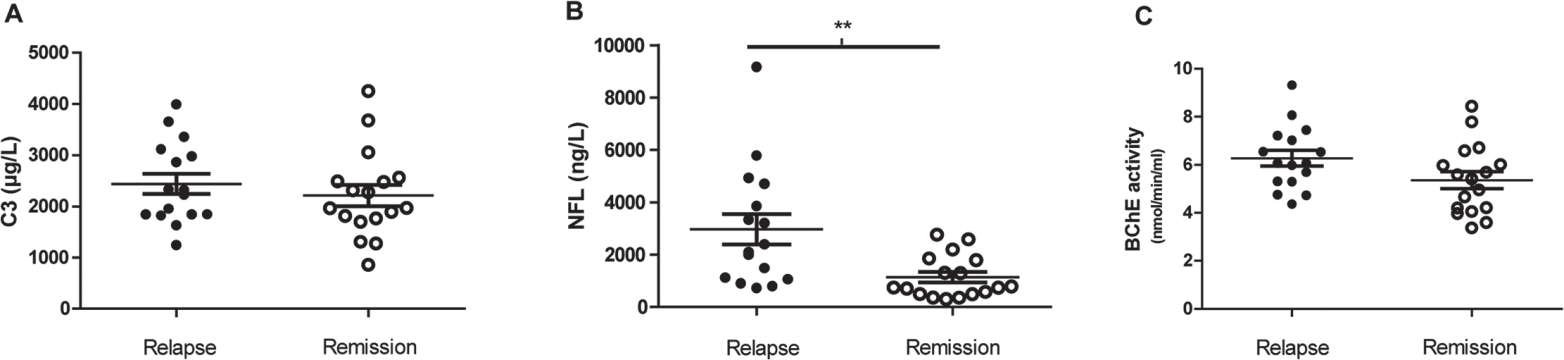
RRMS clinical relapse activity increase CSF NFL levels, but not BuChE activity or C3. RRMS patients in relapse (n = 16) as compared to patients in remission (n = 17). C3 levels were not affected by clinical disease activity in RRMS (A), while NFL levels were significantly higher after relapse (B). For BuChE activity there was a non-significant (p = 0.06) trend for an increase in patients with a relapse. * = p < 0.05; ** = p < 0.01; *** = p < 0.001.

In the RRMS group 16 patients had a clinical relapse within 3 months of sampling, while 17 were in remission. CSF levels of C3 were not different between the two groups (Fig 3). In contrast, and as expected, NFL levels were significantly increased in patients with a recent relapse. BuChE activity displayed a non-significant trend (p = 0.061) for an increase in patients with a relapse as compared to patients in remission (Fig 3).

## Discussion

We here find increased levels of the key complement component C3 in the CSF of MS patients, which correlate with the degree of clinical disability, as well as one of the most established markers of ongoing neurodegeneration, NFL [31–33].

The MS cohort consisted mainly of untreated RRMS patients with active disease and 16 patients were in relapse as defined by a worsening of neurological functions at the time of sampling. The group of RRMS patients was either newly diagnosed or had interrupted treatment due to pregnancy or personal reasons. Only two were on treatment, both RRMS with relapse; one with interferons and the other natalizumab. The levels of NFL we find here correspond well to those we reported earlier at baseline for patients starting natalizumab treatment, with mean NFL levels at 860 ng/L for patients with no relapse activity within 3 months vs 2300 ng/L in patients with a relapse [36]. The corresponding values here were 1129 ± 197 ng/L (n = 17) for patients in remission vs. 2965 ± 576 ng/L (n = 16) for patients with a recent relapse. Generally higher levels of NFL in the RRMS group are also consistent with previous data from our group in a very large cohort of MS patients, showing a negative correlation with age [37]. It is presently unclear to what degree clinical worsening in progressive MS reflects an underlying active inflammatory/neurodegenerative disease process [38, 39]. However, it is clear from the data presented here that at least some patients with progressive disease display elevated NFL levels, suggesting an active disease process. In line with this a recent study demonstrated that immunomodulatory treatments displayed efficacy for lowering CSF NFL levels in progressive patients with increased baseline levels [40].

The finding of increased CSF C3 levels supports the notion of a dysregulation of the complement system in MS and is in line with previous findings in neurodegenerative diseases. Interestingly, and in contrast to NFL, the highest levels were seen in patients with PPMS. Although, this finding should be interpreted with caution due to a small number of patients in the PPMS subgroup, combining overall progressive patients as a group also displayed higher C3 levels as compared to the RRMS group. It is likely that increased levels of NFL in RRMS patients are primarily related to disease activity driven by adaptive immune responses leading to focal areas of inflammation and subsequent axonal injury, whereas a shift towards innate immune activation occurs with progressive disease [6, 41]. Thus, we speculate that increased complement C3, with a potential link to cholinergic activity, is related primarily to more widespread innate inflammatory activation in the nervous tissue. A related observation is that serum levels of factor H, a complement regulatory protein, have been reported to be higher in MS patients with a progressive disease course [10], even if another study found lower CSF levels of factor H in MS patients [14]. Factor H exerts complement inhibitory functions [42], which may suggest increased consumption to balance dysregulated complement activity within the CNS. A previous study showed correlation between intrathecal and serum levels of several complement components, for instance C9 and fB [11]. Levels of C9 were found to be lower in MS patients compared to controls, perhaps illustrating a different aspect of complement activation than studied here.

Accumulating evidence suggests that CNS inherent complement expression may contribute to neurodegenerative processes occurring after various injuries, including inflammation [43–45]. The complement system is complex, with a large number of proteins that together form an intricate biological network regulated at multiple levels, where many of the components have both signalling and direct effector functions [42]. The terminal step of the complement cascade consists of the assembly of the membrane attack complex (MAC), composed of the complement proteins C5b-9. However, the initial part of the cascade can be partially activated without formation of MAC [46, 47]. Due to this complexity it is difficult to speculate to what degree complement activation is an upstream phenomenon contributing to increased neuronal injury or if increased C3 levels merely reflect a downstream effect of a neurodegenerative process driven by other mechanisms. Still, the genetic association between AD risk and the complement related genes clusterin and complement receptor 1 suggests that the complement system can act upstream in the disease process, even if the exact mechanisms remain to be elucidated [48]. Also, the association between increased intrathecal C3 in other neurodegenerative diseases [17] and our findings here with the highest CSF C3 levels in patients with more MRI lesions and in the progressive phases of disease points to potentially important pathways involved in the chronic neurodegenerative phase of MS, which is less understood than its initial relapsing-remitting phase. However, it should be noted that the finding of increased C3 in patients with ≥9 cerebral MRI lesions should be interpreted with caution since they were compared with a substantially lower number of cases with little lesion burden.

In this context, the correlation between CSF levels of C3 and BuChE in MS patients is interesting. In contrast, even if AChE levels differed between MS patients and controls, there was no significant association to C3 at the individual level, arguing against a role for AChE in regulation of C3 expression in MS patients. In contrast to our study, Antonelli et al. did not detect a significant difference in AChE between RRMS and OND [25]. A likely explanation is the choice of method; we measured AChE activity with an optimal concentration of 0.5–1 mM acetylthiocholine (ATCh, an ACh analogue), which has a greater biological relevance. Measurements at lower ATCh/ACh concentrations are less informative and might not completely reflect the full enzymatic capacity of AChE. As mentioned previously a relative increase in BuChE over AChE in the diseased CNS is also supported by previous neuropathological studies [22–24]. Also, recent studies underscore the importance of cholinergic signaling for adaptive immune responses in the periphery [49]. There may also be analogous effects mediated by ACh in the brain, since both astrocytes and microglia express nicotinic cholinergic receptors that attenuate pro-inflammatory responses [50–53]. In addition, a number of studies have reported neuroprotective effects mediated through cholinergic signaling across different experimental models of neuronal injury [54–56]. However, several questions still remain, such as the relative role of AChE and BuChE for regulating cholinergic tone in CNS tissue, and to what degree this balance is important for regulation of inflammation. ACh in biological samples is unstable and degrades spontaneously without addition of cholinesterase inhibitors, making it unreliable as a biomarker. In addition, the local concentration in the tissue may well be different from CSF levels.

BuChE is an enzyme with several functions that are still under investigation but not yet fully understood. For instance, BuChE shows peptidase activity and activates trypsin [57], and shows a major role in the metabolic pathways of many substances that reaches the circulation. In addition, the distribution pattern of this enzyme in the CNS is complex and point at important functions not limited to the classical cholinergic neurotransmission [58]. Thus, we speculate that BuChE, together with a soluble form of the ACh-synthesizing enzyme ChAT is involved in the regulation of extracellular ACh signaling, apart from cholinergic neurotransmission, and thereby regulates the functional status of various cholinoceptive, non-excitable cells, such as astrocytes, microglia and oligodendrocytes; all of which should be of high relevance to MS in context of inflammatory processes in the CNS [34, 59, 60]. We recently also demonstrated that differentiation of neurospheres lead to gliogenesis in synchrony with a reduction of released ChAT, but with increases in BuChE, i.e. causing a microenvironment favoring hypo-cholinergic signaling accompanied with altered cytokine profiles [59, 61].

Adding additional complexity is the possibility of changes in the extra synaptic ChAT as an alternative pathway of regulating ACh levels, as recently reported by us [60]. Our findings here suggest the possibility of a link between BuChE activity and expression of C3, even if it may also be that both are regulated by a common upstream event and therefore not directly related to each other. One such factor could be reduced physical activity as a result of increasing disability. Somewhat unexpectedly we did not find evidence suggesting that C3 or BuChE are regulated by clinical disease activity in the RRMS group. This further strengthens the notion of qualitative differences in the inflammatory milieu between RRMS and progressive patients [5–7].

In conclusion we here demonstrate that the levels of C3 in CSF of MS patients correlate to disease course/severity and a biomarker for nerve injury. This finding provides additional support for the notion of complement dysregulation as an integral part of MS disease processes, especially in later disease stages characterized by increased disability and neuronal injury. The data also support the notion that innate immune mechanisms are relatively more important in later stages of the disease [5–7]. We also report the novel observation of a correlation between CSF C3 levels and BuChE enzymatic activity, which suggests that expression of complement C3 may be influenced by cholinergic tone. Further studies are needed to address the question if increased complement activity reflects an up or down stream event in neurodegenerative processes occurring in MS and the potential regulatory role of cholinergic signaling.
